# Successful Treatment of Long-Term Severe Progressive Interstitial Pneumonia with Low-Dose Corticosteroid and Azathioprine in a Patient with Diffuse Systemic Sclerosis

**DOI:** 10.1155/2012/143927

**Published:** 2012-10-03

**Authors:** Takuya Kotani, Tohru Takeuchi, Shigeki Makino, Toshiaki Hanafusa

**Affiliations:** First Department of Internal Medicine, Osaka Medical College, 2-7 Daigaku-Machi, Takatsuki, Osaka 569-8686, Japan

## Abstract

For progressive interstitial pneumonia (progressive IP) that accompanies diffuse systemic sclerosis (diffuse SSc), no treatment guidelines have yet been established, and it is a complication with a poor prognosis. We herein report a case in which combination therapy of a low-dose corticosteroid and low-dose azathioprine was performed for progressive SSc-IP in a 64-year-old female whose respiratory function was severely damaged for a long period of time and for whom improvement was achieved. The beneficial effect has continued for 3 years with no side effects being observed during the course.

## 1. Introduction

Systemic scleroderma (SSc) is a systemic autoimmune disorder of the gastrointestinal tract, lungs, and kidneys, and so forth, which has dermal sclerosis as a major characteristic. SSc has IP as a complication at a frequency of 44 to 75% [1, 2]. SSc-IP normally progresses slowly, but in diffuse SSc-IP, there are cases in which progression is rapid and also cases in which the respiratory function is damaged for a long period of time [3]. No treatment guideline has been established for progressive SSc-IP. Treatment methods that have been attempted most frequently are oral cyclophosphamide (CY) and intravenous pulse CY (IVCY). Although improvement in respiratory function has been obtained by these methods for a short period of time of 1 to 2 years, they are not easy to be applied for longer periods of time due to serious side effects [4, 5].

Azathioprine (AZA) inhibits purine nucleotide synthesis and exerts an immunosuppressive effect. A report exists in which respiratory function stabilized upon switching treatments to AZA as a maintenance therapy after IVCY was performed monthly for 6 months for SSc-IP in addition to a low-dose corticosteroid [6]. Moreover, a report exists in which combination therapy of a steroid and AZP improved the survival rate of idiopathic interstitial pneumonia [7]. Because AZA has fewer serious side-effects than CY, there is a possibility that it can also be used for SSc-IP for a long period of time.

In this paper, we describe a case in which continuous improvement of IP was observed with combination therapy of a low-dose corticosteroid and AZA for a case of SSc-IP in which the pulmonary damage had inveterately progressed for a long period of 12 years.

## 2. Case Report

**Figure 1:**
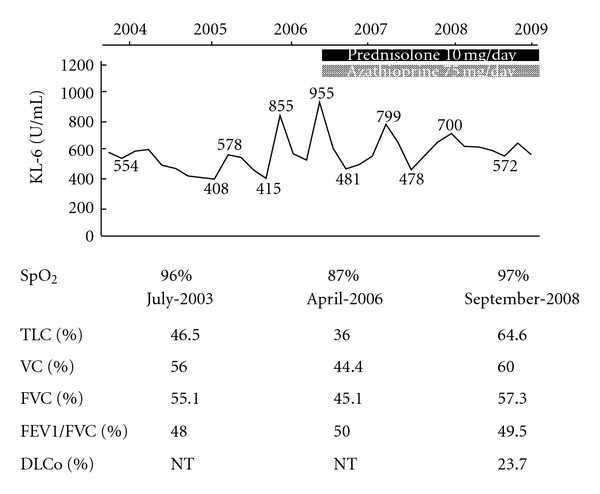
Clinical course. TLC: total lung capacity, VC: vital capacity, FVC: forced vital capacity, FEV1*/*FVC: forced expiratory volume in 1s/forced vital capacity, DLCo: carbon monoxide diffusing capacity, and NT: not tested.

**Figure 2:**
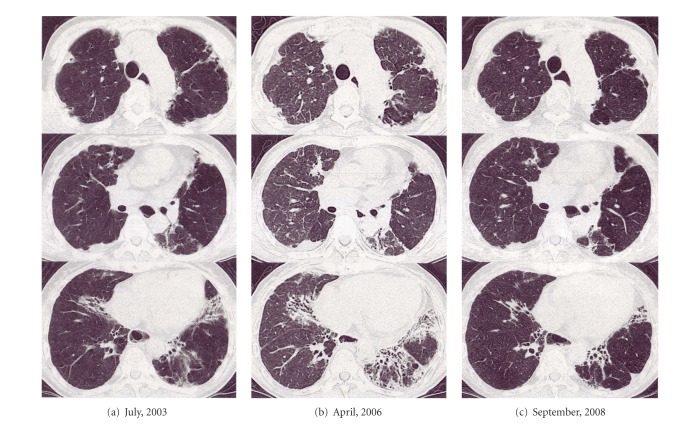
Course of chest HRCT. (a) Consolidations, ground-glass opacities (GGO), and traction bronchiectasis (TBE) were noted on chest HRCT at July, 2003. (b) Consolidations, GGO, and TBE increased on admission in April, 2006, compared with that in April, 2003. (c) Consolidations, GGO, and TBE improved with 10 mg/day of prednisolone and 75 mg/day of azathioprine at September, 2008.

A 52-year-old female was admitted to our hospital for dry cough and exertional dyspnea (Hugh-Jones classification (H-J) class II) on July 1994. High-resolution computed tomography (HRCT) of the chest showed a reticular shadow on the dorsal side of both lower lungs, ground-glass opacity (GGO), and traction bronchiectasis (TBE), and she was diagnosed as having IP. Around January 2000, Raynaud's phenomenon and, in the distal portion of the limbs, dermal sclerosis appeared. The dermal sclerosis diffusely progressed throughout the entire body thereafter, and she was consequently diagnosed as having IP followed by SSc. The clinical course thereafter is shown in Figure 1 and the chest HRCT findings in July 2003 are shown in Figure 2(a). In July 2003, the SpO_2_ was 96% (room air), the KL-6 was 554 U/mL. In a respiratory function test, the TLC% was found to be 46.5% and the FVC% was 55.1%. From October 2005, exertional dyspnea had exacerbated from H-J class II to IV, the SpO_2_ decreased to 87% (room air), and she was admitted to our hospital on April 3, 2006. On physical examination, fine crackles were observed in both lower lungs, and dermal sclerosis was diffusely observed in the facial surface and all limbs. In the laboratory findings, the WBC was 7240/*μ*L (neutrocyte 69.2%) and the CRP was 3.20 mg/dL. The KL-6 was 955 U/mL. The antinuclear antibody was positive (speckled pattern), but the other various disease-specific autoantibodies were negative including anti-Scl-70 antibody. In a respiratory function test, the TLC% decreased to 36.0% and the FVC% to 45.1%. In the chest X-ray, both lower lungs shrank, the reticular shadow increased, and the chest HRCT showed increase in the area of the consolidation, GGO, and TBE (Figure 2(b)). SSc-IP was determined to have exacerbated, and on April 11, 2006, combination therapy of 10 mg/day of prednisolone (PSL) and 75 mg/day of AZA was initiated. The IP gradually improved, and in September 2008, the dyspnea improved to H-J class I and the SpO_2_ increased to 97%. The KL-6 decreased to 572 U/mL, and in a respiratory function test, the FVC% showed improvement to 57.3%. Upon chest HRCT, decrease of the GGO, consolidation, and TBE were observed (Figure 2(c)). During the course, no obvious side effects due to the concomitant therapy of PSL and AZA were observed.

## 3. Discussion

For progressive SSc-IP, the treatment methods most often used are oral CY and IVCY, and it has been reported that respiratory function improves or stabilizes during a follow-up period of 1 to 2 years [4]. However, upon long-term followup, the presence of relapse cases after treatment, an unimproved survival rate, and serious side effects such as hematuria, infertility, bone marrow suppression, malignancy, and infection are problems, in addition to CY not being easy to use for SSc-IP for a long period of time [5].

AZA inhibits purine nucleotide synthesis and exerts an immunosuppressive effect mainly centering on T cells. A report exists indicating the involvement of CD8^+^ T cells with formation of the clinical state of SSc-IP [8] and there is a possibility that AZA is effective for SSc-IP.

In the case reported herein, progressive SSc-IP over a long period of approximately 12 years improved with combination therapy of low-dose PSL and AZA. During the follow-up period of 30 months after the combination therapy, there was no serious side effects, thus resulting in sufficient tolerability. Hoyles et al. reported that AZA was effective as a maintenance therapy for SSc-IP after combination therapy of steroids and IVCY [6]. Dheda et al. have performed combination therapy of low-dose steroids and AZA for 11 cases of SSc-IP and reported that the effects of “improved” (FVC% improved by 10% or more than the base line) in 5 patients and “stable” (FVC% improved by 10% or less) in 3 patients were obtained [9]. When the 8 cases of Dheda et al. and the present case are compared, the 8 cases of Dheda et al. showed a mean duration for IP of 18.45 ± 4.52 months and a mean FVC% of 54.25 ± 3.53%, whereas the present case showed a duration for IP of 171 months and a FVC% of 45.1%, and is a case in which the respiratory function was damaged for a much longer period of time. As in our case, there is a possibility that even SSc-IP in which the respiratory function was severely damaged for a long period of time may improve with combination therapy of a low-dose steroid and AZA, and it is believed to be worth attempting.

In the present case, no obvious side effects due to steroids and AZA were observed. AZA has fewer serious side effects than CPA and there is a possibility that it can be used for a long period of time. However, AZA may also possibly cause side effects such as digestive symptoms, bone marrow suppression, hepatic damage, and increased susceptibility to infection although less severe than those of CY. Dheda et al. have also reported that among the 11 cases of SSc-IP in which AZA was used, side effects of nausea in 1 case and decreased WBC in 1 case were observed, and, therefore, it is necessary to pay attention to the expression of side effects in using AZA for SSc-IP.

As in the present case, combination therapy of a low-dose steroid and AZA is also effective for cases of SSc-IP in which the respiratory function has been severely damaged for a long period of time, indicating that it was a treatment method that could be used for a long period of time. However, because there are still only a few cases in which AZA is used for SSc-IP, it is necessary to evaluate the efficacy and safety after the future accumulation of more cases.
